# Shadows of very high-frequency oscillations can be detected in lower frequency bands of routine stereoelectroencephalography

**DOI:** 10.1038/s41598-023-27797-9

**Published:** 2023-01-19

**Authors:** Zuzana Vasickova, Petr Klimes, Jan Cimbalnik, Vojtech Travnicek, Martin Pail, Josef Halamek, Pavel Jurak, Milan Brazdil

**Affiliations:** 1 Brno Epilepsy Center, Department of Neurology, St. Anne’s University Hospital, Faculty of Medicine, Masaryk University, Brno, Czech Republic; 2 International Clinical Research Center, St. Anne’s University Hospital, Brno, Czech Republic; 3 Institute of Scientific Instruments, The Czech Academy of Sciences, Brno, Czech Republic; 4 Behavioral and Social Neuroscience Research Group, CEITEC Central European Institute of Technology, Masaryk University, Brno, Czech Republic

**Keywords:** Epilepsy, Computational neuroscience

## Abstract

Very high-frequency oscillations (VHFOs, > 500 Hz) are more specific in localizing the epileptogenic zone (EZ) than high-frequency oscillations (HFOs, < 500 Hz). Unfortunately, VHFOs are not visible in standard clinical stereo-EEG (SEEG) recordings with sampling rates of 1 kHz or lower. Here we show that “shadows” of VHFOs can be found in frequencies below 500 Hz and can help us to identify SEEG channels with a higher probability of increased VHFO rates. Subsequent analysis of Logistic regression models on 141 SEEG channels from thirteen patients shows that VHFO “shadows” provide additional information to gold standard HFO analysis and can potentially help in precise EZ delineation in standard clinical recordings.

## Introduction

Epilepsy is a severe chronic neurological disorder that is mostly well-controlled using anti-seizure medication (ASM). Long-term seizure freedom can be achieved in the majority of patients. Unfortunately, despite considerable recent developments in ASM and well-adjusted pharmacological treatment, 30–40% of all patients remain drug-resistant with ongoing seizures^1^. Consequently, epilepsy surgery (either resective or neurostimulation) should always be considered to eliminate seizures in drug-resistant subjects suffering from focal epilepsy^2^. Nowadays, the best practice represents performing a curative resective surgical procedure aiming to remove brain tissue responsible for seizure genesis—the epileptogenic zone (EZ). To localize and delineate EZ, intracranial EEG (iEEG) is performed in selected patients eligible for resective epilepsy surgery^3^. Although iEEG is a powerful tool for the identification of irritative zone (IZ—brain regions producing interictal epileptic spikes) and actual seizure onset zone (SOZ—brain region with the very first ictal change of the EEG pattern), it is still somewhat limited in precise delineation of EZ which remains to be a grossly theoretical concept^4^. Importantly, the removal of SOZ is mostly, but not always resulting in a long-term seizure-free outcome^5^.

With EEG advances, it is currently possible to monitor brain activity with frequencies over 100 Hz. The recordings with amplifiers using high sampling frequency allow us to investigate interictal high-frequency oscillations (HFOs) which are EEG activities in the frequency range 80–500 Hz^6^. In combination with interictal spikes, HFOs have high specificity in indicating the SOZ^7,8^, and removal of interictal HFO-generating areas correlated with good postsurgical seizure outcomes^6,9–12^. Thus it seems that HFOs contain information that can optimize the diagnosis and treatment of epilepsy. However, using HFOs in an individual prognostication of seizure outcome is correct in 67% of patients only^7^. Recent studies suggest that within mesiotemporal regions, interictal very high-frequency oscillations (VHFOs) with frequencies over 500 Hz are more specific biomarkers in localizing SOZ, and possibly even EZ, than interictal HFOs or spikes^13,14^.

In spite of technological advancement, recordings with extremely high sampling frequencies that are necessary for VHFO identification are still too demanding and expensive to be routinely used in standard clinical settings. The aim of the present study is to investigate whether “shadows” of the VHFOs can be captured in the EEG containing frequencies up to 450 Hz. We hypothesized that it is possible to distinguish iEEG signals/channels with or without the presence of VHFOs, using recordings with a reasonable sampling frequency of 1 kHz.

## Results

### Patients

**Figure 1:**
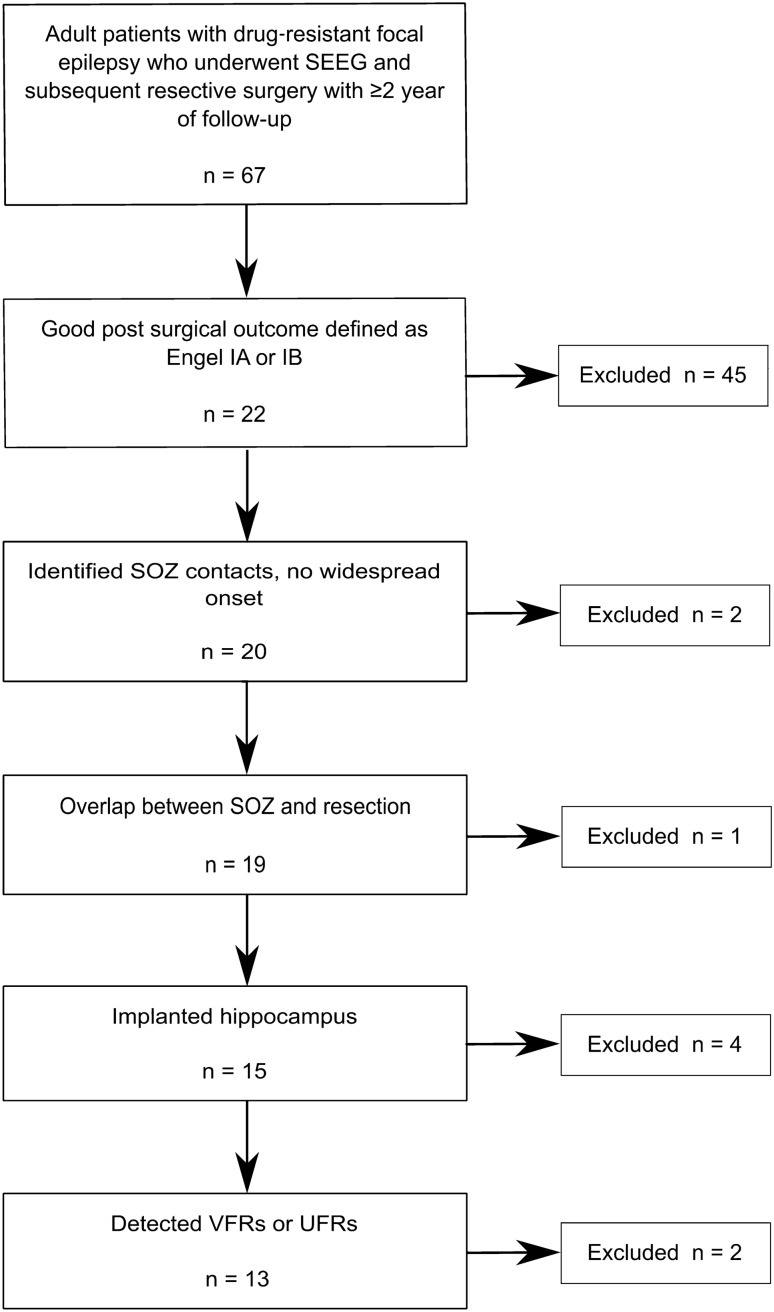
Patient selection flowchart.

The whole database of all SEEG patients consisted of 103 patients. Two patients were not adults at the time of the surgery, 14 had non-resective surgery or were not operated on at all and 20 patients did not have a 2-year post-surgical outcome. The initial cohort consisted of 67 patients. 45 patients had poor outcome > Engel IB and were excluded in order to analyze only channels which resection would be valuable, ensuring that the proposed methods would aid better postoperative results. Consequently, two patients did not have clearly localized SOZ, one patient had no overlap between SOZ and resection, four patients were not implanted in the hippocampus, and in two patients no VHFOs were detected. The selection flowchart is shown in Fig. 1. In the final 13 selected patients (8 females, age 36.63 ± 12.10; 5 males, age 40.40 ± 5.99), data from 141 contacts positioned in the hippocampus or uncus gyri parahippocampalis or amygdala was investigated, as described in Table 1.

**Table 1:** Characteristics of selected 141 contacts from 13 patients and identification of contacts with ripples, FR, VFR, and UFR. Patient 21 had contact C1 on the edge of the resection (< 0.5 mm); patient 26 had the resected contacts contralateral side; patient 40 had contact C1 on the edge of the resection (< 0.5 mm); patient 45 had UFRs present only in short sections. A/A′—amygdala (pts. 003, 004, 026, 040, 045), uncus gyri parahippocampalis (pts. 013, 071), B/B'—hippocampus (pts. 031 (B′1–B′4))/ventral hippocampus (pts. 003, 004, 013, 015, 021, 026, 031 (B1–B3), 033, 040, 045, 047, 071, 082)/parahippocampal gyrus, C/C′—dorsal hippocampus, P—uncus gyri parahippocampalis, L—fusiform gyrus (pt. 082)/middle temporal gyrus (pt. 021 (L8–L10))/lesion (pt. 021 (L1–L4)), H—fusiform gyrus/lower temporal gyrus, T′—upper temporal gyrus, Tp/Tp′—temporal pole (pt. 031)/parahippocampal gyrus (pt. 045 (Tp1–Tp2))/transversal temporal gyri (pts. 040, 045 (Tp3–Tp10)), ′ indicating prime side.

Patient no.	SOZ	Resection	Selected Contacts	Ripples	FR	VFR	UFR
3	B′1–2	A′, B′1–5, C′1–3	A′1–3, B′1–4, C′1–3	A′1–2, B′1–4, C′1	B′1–3	B′1	–
4	B1–3, C1	A, B1–5, C1–3	A1–3, B1–4, C1–3, A′1–3, B′1–3, C′1–3	A1–3, B1–3, C1, C′1–2	A1–3, B1–3	A1–2, B1	–
13	A′5–6	A′, B′1–7	C′1–3, B1–3, B′1–3, A′1–2	A′1–2, B1–3, B′1–3, C′1–3	B′1–3, C′1–2	B′1–3	–
15	B′1–4, C′1–4	B′1–3	A′1–3, B′1–4, C′1–4	B′1–4, C′1–4	B′1–2, C′1–3	B′1–2, C′2	–
21	B1–4, C1–3	L, A1–4, B1–4	A1, B1–4, C1–3, A′1, B′1–3	B3–4, B′1–3, C1–3	B3–4, B′1–3, C1–3	B3–4, C1–2	C1
26	B′1–3	A′, B′, T', C′7–10	B2–4, C2–3, A′1–3, B′1–3, C′1–3	A′1–2, B′1–2, C′1–2, B2–4, C2–3	B12–3, B′1–2, C2–3, C′1–2	B2–3, C2	–
31	B′1–4, C′1–4, B1–3	Tp′, A′, B′1–5	A1–3, B1–3, A′1–4, B′1–4, C′1–4	B′1–4, B1–3, C′1–3	B′1–4, B1–2, C′1–3	B′1–3	–
33	B1–2	Tp, A, B1–7	A1–2, B1–3, C1–2, B′1–3, C′1–4	B1–3, C1–2, C′1–4, B′1–3	B1–3, C1–2	B1–2	–
40	Tp, B1–3, C1–3	Tp1–5, B, A6–10	A1–3, B1–3, C1–3, A′1–3, B′1–2, C′1–3	A1–3, B1–3, C1–3, A′2, B′1–2, C′1–2	A1, B1–3, C1–3, A′2, B′1–2, C′1–2	B1–2, C1–2	B1–2, C1
45	B1–3	Tp, B1–4	A1–3, B1–3, C1–3, B′1–3, C′1–3	A1–3, B1–3, B′1–3, C1–3, C′1–3	B1–3, B′1–3, C1–3, C′1–3	B1–3, B′1–3, C1–2, C′2	B1–3, B′1
47	B1–3	A, B1–5	A1–3, B1–3, C1–3, B′1–2, C′1–2	B1–3, C1–3, C′1–2	A1–3, B1–3, C1–3,	B1–3	B1
71	C′1–4, B′1–5	A′, B′1–6	A1–2, B1–5, A′1–2, B′1–4, C′1–4	A1–2, A′1–2, B1–5, B′1–4, C′1–3	A1–2, A′1, B1–5, B′1–4, C′1–3	B1–5, B′1, C′1–2	B′1, C′1
82	B1–3, P1–2, L1–6, H1–4, C1–4	P, B, L1–6	B1–3, C1–5	B1–3, C1–4	B1–3, C1–4	B1, C1–2	C1

### SEEG channels manual classification

Raw SEEG recordings with a sampling rate of 5 kHz were manually checked for the presence of HFO and VHFO. Channels with HFOs and VHFOs were marked as VHFO channels. Channels with HFOs in ripple (R) or fast ripple (FRs) range and without the presence of VHFOs were marked as HFO channels. Channels without any HFO activity were removed from further processing. Consequently, 50 VHFO SEEG channels and 91 HFO SEEG channels were downsampled to 1 kHz and the following features were calculated: Amplitude Maximum, 75th Percentile of power spectral density, Shannon Entropy, and Teager–Kaiser Energy Operator (TKEO).

### Differences between VHFO and HFO channels

**Figure 2:**
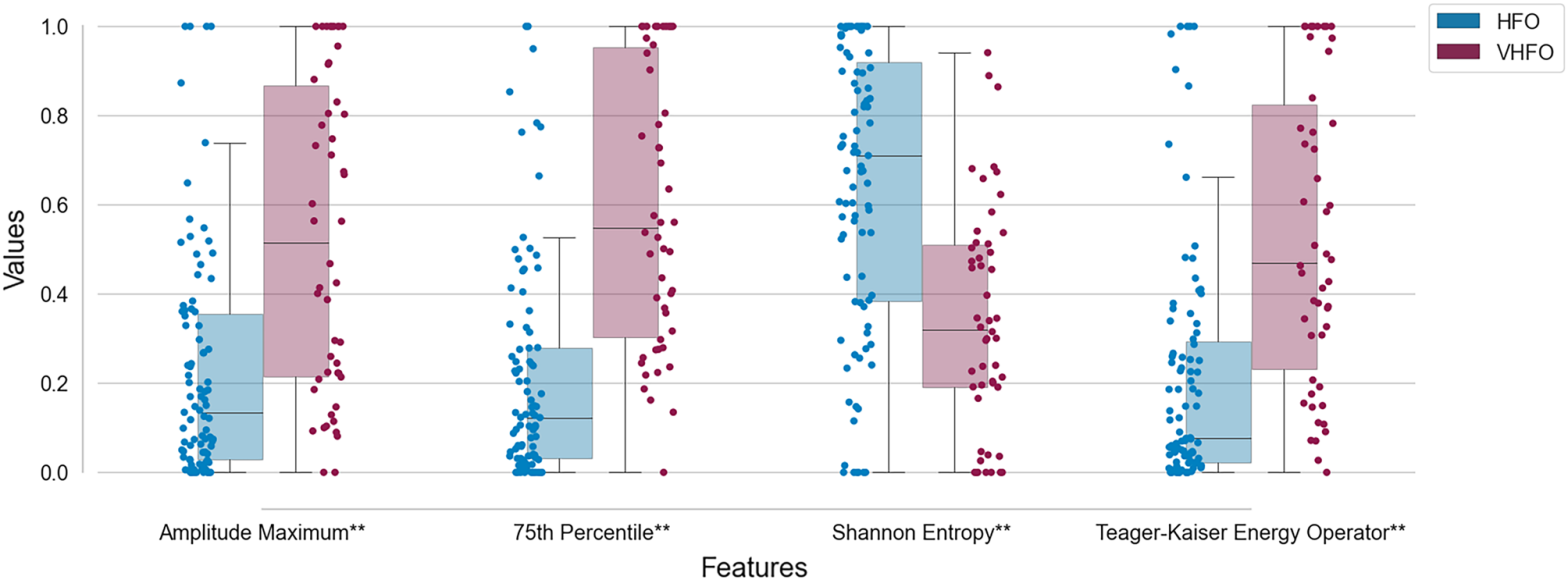
Boxplots of features of HFO and VHFO channels with 1 kHz sampling frequency. The boxes show the quartiles of the dataset while the whiskers extend to show the rest of the distribution. Outliers are not visualized. **Results with p ≤ 0.01 and large Cliff’s delta are considered significant.

There were significant differences between VHFO and HFO channels in all of the features calculated from undersampled 1 kHz SEEG channels (Fig. 2). Calculated p values after Bonferroni correction and effect sizes are concluded in Table 2. When filtered in different traditional EEG frequency bands, the results show significant differences between VHFO and HFO channels in frequencies mainly above the Theta band. The p values after Bonferroni correction calculated in 7 different frequency bands are summarized in Table 3.

**Table 2:** Statistics calculated for features of HFO and VHFO channels with 1 kHz sampling frequency with Bonferroni correction.

Feature	p Value	Cliff's delta	Cliff's delta interpretation
Amplitude maximum	0.0001	0.5305	Large
75th percentile	< 0.0001	0.6888	Large
Shannon entropy	0.0002	− 0.5248	Large
TKEO	< 0.0001	0.5824	Large

**Table 3:** p values calculated for features of HFO and VHFO channels with 1 kHz sampling frequency in different frequency ranges (delta (0.5–4 Hz); theta (4–7 Hz); alpha (8–12 Hz); beta (14–30 Hz); lower gamma (31–45 Hz); upper gamma (55–80 Hz); ripples (80–200 Hz). Results with p ≤ 0.01 and large Cliff’s delta are deemed as significant (*) and the rest as ns = non-significant.

Feature	Delta	Theta	Alpha	Beta	Lower Gamma	Upper Gamma	Ripples
Maximal absolute amplitude	ns	0.0394	< 0.0001*	0.0002*	< 0.0001*	< 0.0001*	< 0.0001*
75th percentile	ns	ns	0.0013*	0.0049*	0.0002*	< 0.0001*	< 0.0001*
Shannon entropy	ns	ns	0.0106*	ns	ns	0.0055*	0.0001*
TKEO	ns	ns	0.0012*	0.0044*	0.0001*	0.0004*	< 0.0001*

### HFO rates

**Figure 3:**
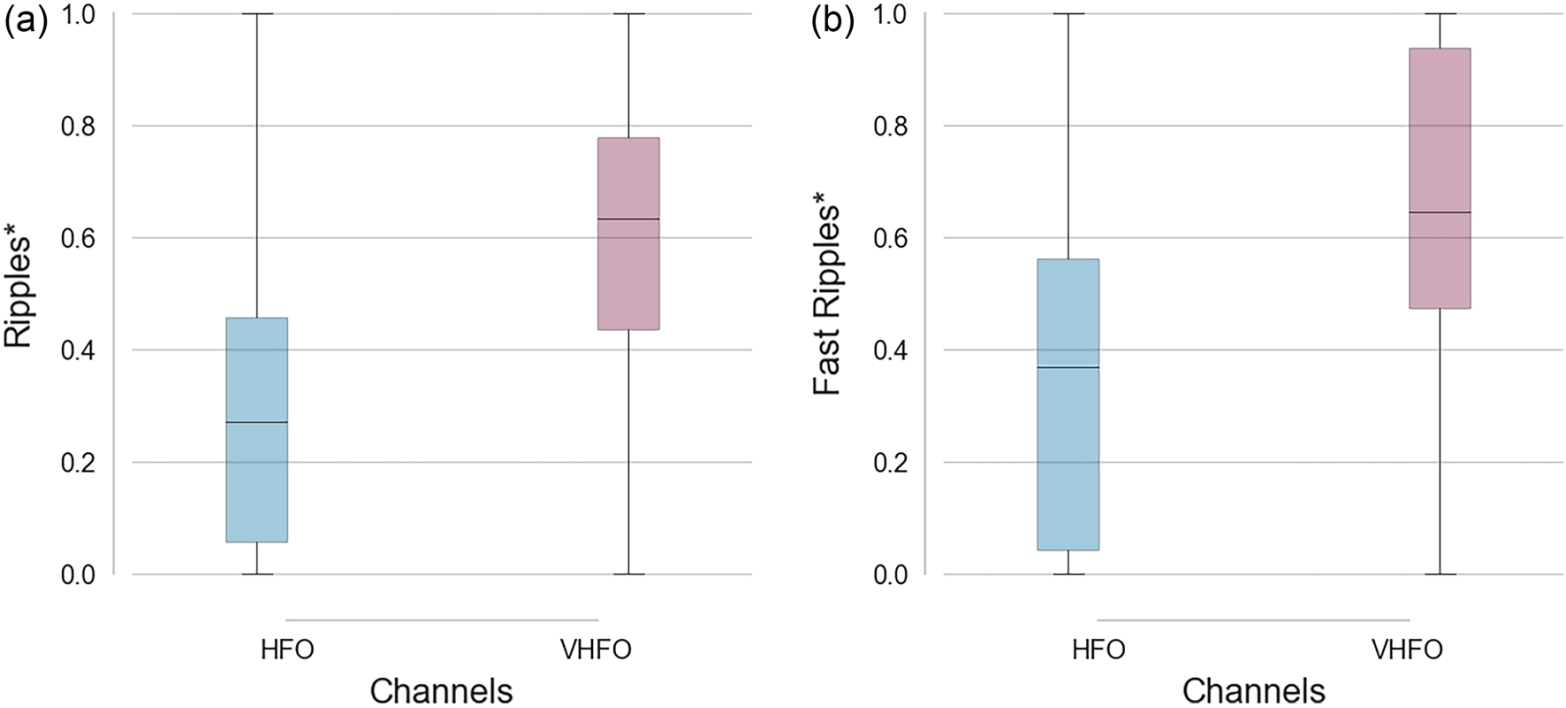
(**a**) Boxplot of ripple rates detected from 5 kHz data in HFO and VHFO channels. (**b**) Boxplot of fast ripple rates detected from 5 kHz data in HFO and VHFO channels. *Results with p ≤ 0.01 and large Cliff’s delta were considered significant.

Furthermore, statistically significant differences were proven between the ripple rates of VHFO and HFO channels (p = 0.0007, large Cliff’s delta = 0.4798) and between the fast ripple rates of VHFO and HFO channels (p = 0.0001, large Cliff’s delta = 0.5176) calculated from data with sampling frequency 5 kHz (Fig. 3).

### Logistic regression

The accuracies and F1 scores of logistic regression models trained on ripple rates detected on 5 kHz data (Model 1), described features (Model 2), and HFO rates detected on 5 kHz and features combined (Model 3) are summarized in Table 4. The p-values of the Wilcoxon rank-sum test after Bonferroni correction between accuracies and F1 scores are significant for Model 1 and Model 2, and Model 1 and Model 3, while the p values are insignificant for Model 2 and Model 3.

**Table 4:** Accuracies and F1 scores of logistic regression models trained respectively on HFO rates detected on 5 kHz data (Model 1), calculated features (Model 2), and a combination of the previous (Model 3).

	Model 1	Model 2	Model 3
Accuracy	0.7026 ± 0.0606	0.8005 ± 0.0614	0.8378 ± 0.0470
F1 Score	0.8246 ± 0.0350	0.8816 ± 0.0363	0.9035 ± 0.0284

## Discussion

Recently two independent research groups published influential studies suggesting an extraordinary role of VHFOs (500–2000 Hz) in human SEEG recordings for more precise delineation of the epileptogenic zone. These oscillations seem to be preferentially observed within mesiotemporal regions and were promisingly described as more specific biomarkers than ripples and FRs^13,14^. Similarly to red and green spikes, where the red spike zones are recognized as zones generating seizures and the green spike zones are recognized as zones generating only sporadic, isolated, spatially confined discharges^15^, the examined channels could be divided into two groups of VHFO and HFO channels based on the VHFO information. The division could possibly resolve which HFOs are physiological, seemingly the HFO channels, and which HFOs are not physiological—the VHFO channels. However, high-quality, high-resolution, and low-noise recordings are critical for the identification of VHFOs. These requirements usually cannot be fulfilled in traditional clinical settings and, therefore, practical use of VHFO detection in clinical practice can be hardly expected in the near future. Thus, a question arose if the presence of VHFO could be estimated from standard SEEG recordings (sampled at 1 kHz) utilizing any other specific feature/s.

Our study investigated 1 kHz SEEG recordings, manually assessed in section Methods—SEEG channel selection and manual classification as with/without VHFOs, for shadows of VHFOs in lower frequencies, focusing on the possibility of determining their presence or absence.

This study included 141 SEEG recordings, from a cohort of 13 patients, undersampled from 5 to 1 kHz with a focus on VHFOs. 126 out of the total 141 (89%) of the contacts with present VFRs and/or UFRs were contacts that were also resected in the surgery. Contacts that were not resected were found in four patients: patient 15 contact C′2, patient 26 contacts B1–3, C1–2, patient 71 contacts B1–5 and C′1–2, and patient 82 contacts C1–2 (Table 1). Even if the contacts were not resected they were still used in the analysis and are included in the results.

Our results suggest that based on the calculated features it is feasible to differentiate between HFO and VHFO channels even in lower sampling frequencies. The calculated features, Maximum amplitude, Shannon entropy, and TKEO were calculated in the time domain of a signal while the 75th percentile was calculated from power spectral density estimates proposing that VHFOs to some extent directly affect the power spectrum below 450 Hz of the recorded signal. When the recordings were filtered into seven distinctive frequency bands, bands above theta also show significant differences in Amplitude Maximum, 75th percentile, and TKEO features, suggesting that VHFO activity affects the signal recording in certain frequency bands. On the contrary, Shannon Entropy is non-significant in most of the seven respective ranges, providing significant values in theta, alpha, and ripple bands. Shannon Entropy is expressing a value unrelated to the remaining features, describing the information and variability of the signal. Filtered EEG signal becomes more deterministic and causes the entropy to lower even in HFO channels, which have significantly higher Shannon Entropy than VHFO channels, causing the medians of these two groups to overlap after the filtration into narrow frequency ranges. (Table 3, Supplement Fig. 1).

Alternatively, the significant differences could be justified by the presence of HFO—ripples and fast ripples. The analysis of HFO rates and the non-significant p values validates that the HFO rates detected on the original 5 kHz are sufficient to differentiate between HFO and VHFO channels. Accuracies of the three trained models confirm that the proposed features bring new information compared to rates of HFOs only as they improve with the inclusion of the proposed features. The model trained on the calculated features and HFO rates has the best accuracy and F1 score, while the model trained in HFO rates has lower accuracy and F1 score than the model trained on feature values. The p-value of the Wilcoxon rank-sum test after Bonferroni correction between accuracies of proposed models describes significant differences between accuracies of Model 1 and 2, and Model 1 and 3, and no significant differences were confirmed between accuracies of Model 2 and 3. To conclude, shadows of VHFOs are present in lower frequencies of the SEEG recordings. The results suggest that it is possible to distinguish SEEG signals with or without the presence of VHFOs, using recordings with a sampling frequency of 1 kHz. The differences between the accuracies and F1 scores also suggest that our features provide additional information to traditional ripple and fast ripple rates. Introducing a computation of Amplitude Maximum, 25th Percentile, Shannon Entropy, and Teager–Kaiser energy operator into the analyses of SEEG recordings with standard sampling frequency improves our ability to differentiate HFO and VHFO channels. The determined VHFO channels could facilitate the resection and potentially improve epilepsy surgery outcomes^14^.

## Methods

### Patients

The selected patients were all adults with drug‐resistant focal epilepsy who underwent stereoelectroencephalography (SEEG) and subsequent resective surgery with ≥ 2 years follow-up at the St. Anne's University Hospital since 2012 when we started with SEEG recordings at a 25 kHz sampling frequency. Inclusion criteria for our study were: (a) good post-surgical outcome defined as Engel IA or IB^16^; (b) identified SOZ contacts; (c) overlap between SOZ and resection; (d) hippocampus, parahippocampal gyrus, or amygdala implanted; (e) detected VHFOs in selected SEEG electrodes. The study was approved by the St. Anne’s University Hospital Research Ethics Committee and the ethics committee of Masaryk University. The study was carried out in accordance with relevant guidelines and regulations. All patients signed an informed consent form.

### SEEG recordings

For depth SEEG recordings, standard intracerebral multi-contact platinum electrodes (5, 8, 10, 12, 15, and 18 contacts) with a diameter of 0.8 mm, a contact length of 2 mm, an intercontact distance of 1.5 mm, and a contact surface area of 5 mm^2^ were used in all patients. Each patient received 5–13 orthogonal SEEG electrodes in the temporal and/or frontal, parietal, and occipital lobes using the stereotaxic coordinate system of Talairach^17^. Their position within the brain was verified using MRI with electrodes in situ. A 192-channel research EEG acquisition system (M&I; BrainScope, Prague, Czech Republic) was used for recording 30 min of awake resting interictal EEG recordings with a sampling rate of 25 kHz and dynamic range of 625 mV with 10 nV (24 bits) and downsampled to 5 kHz with a 2 kHz frequency band. The EEG acquisition unit was battery-powered to reduce line noise. No special shielded environment was used. Thirty minutes of artifact-free continuous interictal SEEG data (recorded during wakefulness) was analyzed for each subject. All recordings were acquired using a referential earlobe reference. For analysis, the signals were subtracted from an averaged signal from all SEEG signals. All data processing and statistics were performed using open platform software SignalPlant^18^ and a Python environment.

### Preprocessing

#### Artifact elimination

To detect artificial segments in the individual recordings, SignalPlant visualization of raw signals simultaneously with Power Distribution Matrices (PDMs) was used^18^.

Artifact detection is crucial for the computation of numerical parameters and statistical evaluation. PDMs provide a complete overview, including artificial signals. SignalPlant visualization of the raw signal simultaneously with PDMs was used to manually identify noisy contacts or time periods with artifacts. Artifacts were detected in each frequency band in PDM format. The number of detected artifacts was the highest in the VFR and UFR bands. Artificial segments and contacts were removed from further processing. The same set of all deselected contacts was used for the R, FR, VFR, and UFR bands^14^.

#### SEEG channel selection and manual classification

Firstly, channels that were located in anatomical structures outside the hippocampus, parahippocampal gyrus, or amygdala, were eliminated from the analysis and were not further considered for visual identification.

The remaining channels were examined for the presence of HFOs and VHFOs (VFRs/UFRs) using visual inspection of graphical PDMs filtered in four different frequency bands (ripples, 80–200 Hz; fast ripples, 200–500 Hz; very fast ripples VFR, 500–1000 Hz; and ultrafast ripples UFR, 1000–2000 Hz). Referred 2-dimensional PDMs consist of rows corresponding to smoothed power estimates (PEs) of respective recorded contacts and of columns corresponding to time intervals. The PDMs were calculated according to the algorithm described in^14^. Based on the reviewed PDMs, 141 channels were selected as channels that showed elevated PEs in ripple and fast ripple frequency bands. These channels’ PEs were subsequently analyzed in VFR and UFR frequency bands. If the channel’s PE was elevated above other channels’ PEs, the channel was subsequently inspected in 5 kHz raw data format for the occurrence of visible oscillations. Identification of contacts with HFOs and VHFOs was blind, without any a priori knowledge of the resected areas and postsurgical outcome (Z.V., P.K., P.J.).

#### HFO rates

An automated HFO detection based on a line-length algorithm^19^ with the threshold set to 6 was used to estimate the HFO rates in all selected channels. The patient HFO rates were min–max normalized and then compared in HFO and VHFO channels in order to test whether it is possible to differentiate between these two groups of channels solely on the information on the HFO rates. The HFO rates were detected in the original signals with a sampling frequency of 5 kHz. HFO rates were not detected on 1 kHz data as it is not feasible to reliably detect FR rates due to sampling frequency constraints.

#### Signal processing

For further processing, all recordings were filtered in the 0–450 Hz range and downsampled from 5 to 1 kHz. Subsequently, multiple features were calculated in a 10-s sliding window moving through the 30 min long recording with an overlap of 50%. The single feature value representing the 30 min long patient channel recording is a median of values of all windows of the recordings. All of the features were calculated on downsampled signals with 0.5–450 Hz frequency band as well as in traditional frequency bands: delta (0.5–4 Hz); theta (4–7 Hz); alpha (8–12 Hz); beta (14–30 Hz); lower gamma (30–45 Hz); upper gamma (55–80 Hz); ripples (80–200 Hz). The frequency range 45–55 Hz was omitted on purpose to avoid the negative effects of the power line hum.

### Calculated features

#### Amplitude maximum

Absolute instantaneous maximum of the signal’s amplitude was estimated using an envelope determined by Hilbert’s transform as envelope = abs(H(x)), where abs is the absolute value, and the final maximum values were calculated as max(abs(envelope))^20,21^.

#### Shannon entropy

Shannon (information) entropy was developed as a unified definition of information-theoretic divergence. It is one of the applicable entropy measures that measure the spread of the data and describe the variability in the EEG signal. High values correspond with a flat probability distribution (typical for noise or random signal) and on the other hand, low values correspond with a narrow probability distribution (typical for deterministic signal). Shannon entropy is defined as H = − sum(p(i)log2(p(i))), where the p are probabilities of a raw signal datum being in a histogram bin^22,23^.

#### Teager–Kaiser energy operator

Teager–Kaiser energy operator (TKEO) is a nonlinear operator determining the instantaneous energy of a non-stationary signal. TKEO is for discrete signal defined as Ψ(x(n)) = x^2^(n) − x(n + 1)x(n − 1), where x is the signal value and n is the sample number^24,25^.

#### 75th Percentile

The 75th percentile is a statistical value of power spectral density estimate. In order to estimate the power spectral density, a nonparametric method of Welch periodogram was used. Welch’s method consisted of dividing the 5-min time series window into segments of length L = 256 with 50% overlap covering the entire window. Afterward, a modified periodogram for each of these segments is calculated, and periodograms for each segment are obtained using the finite Fourier transform. The final spectral estimate of the 5-min window is the average of these periodograms^26^. The final feature value is the 75th percentile of this power spectral density estimate.

### Statistical analyses

Analysis of the relationship between HFO and VHFO channels was carried out by Wilcoxon rank-sum test for two samples of min–max patient-wise normalized data. As an additional statistic, Cliff’s delta calculated the effect size. Wilcoxon rank-sum test for two samples and Cliff’s delta statistics were also used to describe the relationship between HFO ripple and FR rates. Results with p ≤ 0.01 and large Cliff’s delta were considered significant.

### Logistic regression

To test whether our proposed features can differentiate between HFO and VHFO channels and whether they provide additional information to traditional HFO rates, three different logistic regression models were created using all available channels in a cross-validation procedure with 3 splits and 15 repeats. Model 1 used two features: normalized ripple and fast ripple rates detected from 5 kHz recordings. Model 2 used 4 features: Amplitude Maximum, 75^th^ Percentile, Shannon Entropy, and TKEO, calculated from undersampled 1 kHz recordings.

Finally, Model 3 used 6 features: combining ripple and fast ripple rates from 5 kHz data with the calculated features from 1 kHz data. Mean accuracy was calculated for each model separately.

Additionally, the Wilcoxon Rank-sum test of the differences between the accuracies of the proposed models was applied to each possible model pair in the two sets of models in order to test if the difference in accuracies between models was significant.

## Supplementary Information

Supplementary Figures.

## Data Availability

Data is available upon reasonable request to the corresponding author.
