# Supplementary material for: Shadows of very high-frequency oscillations can be detected in lower frequency bands of routine stereoelectroencephalography
Source: Sci Rep. 2023 Jan 19;13:1065. doi: 10.1038/s41598-023-27797-9 (PMC9852423; doi:10.1038/s41598-023-27797-9)
Supplement: Supplementary file 1 — Supplementary Figures. [file 41598_2023_27797_MOESM1_ESM.pdf]

Shadows of very high-frequency oscillations can be detected in lower frequency bands of routine stereoelectroencephalography

Zuzana Vasickova, Petr Klimes, Jan Cimbalnik, Vojtech Travnicek, Martin Pail, Josef Halamek, Pavel Jurak, Milan Brazdil

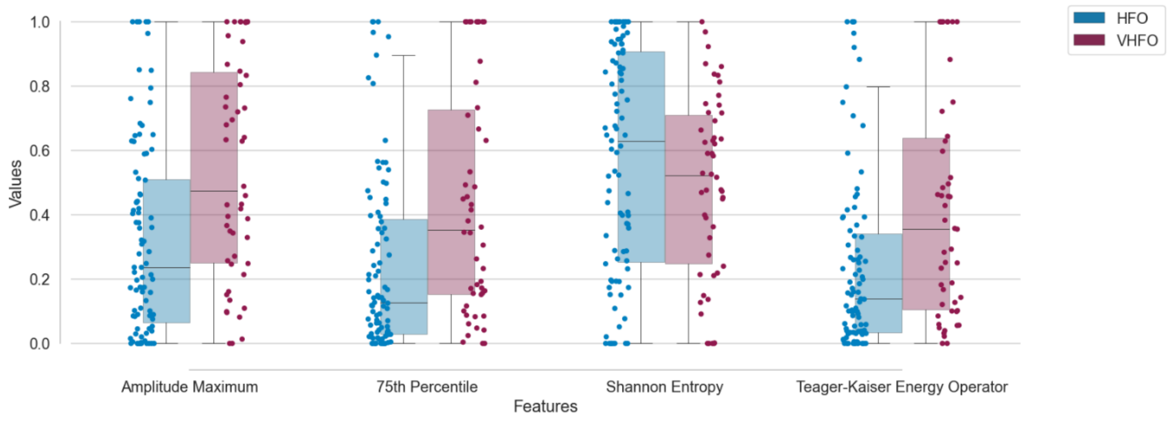

a

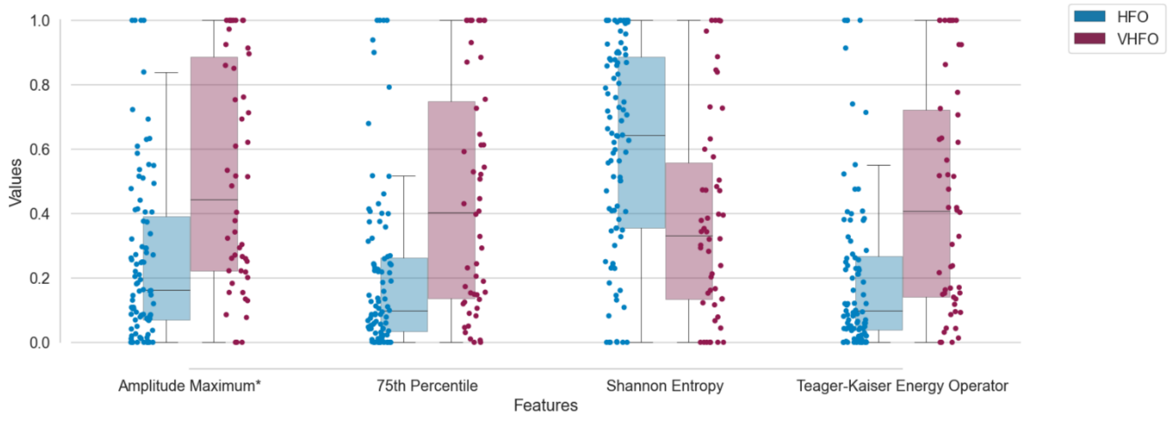

b

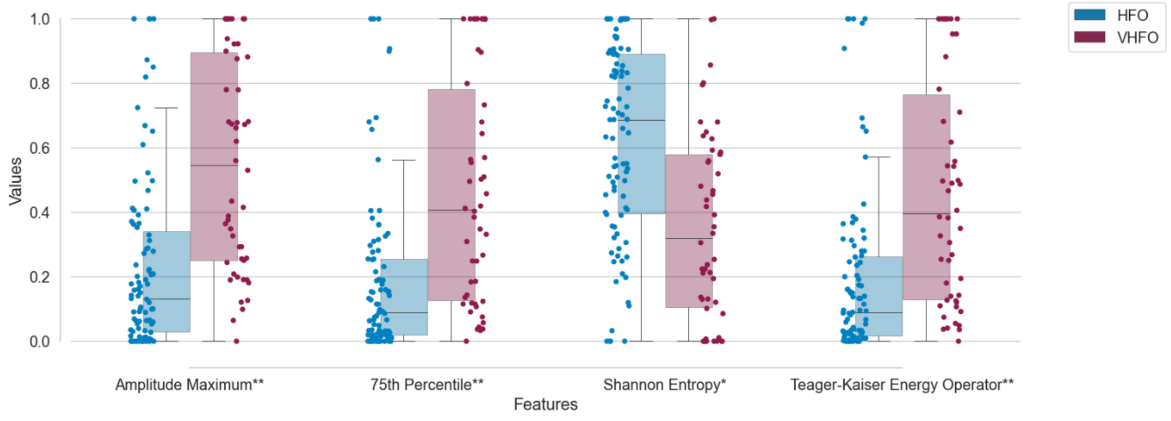

c

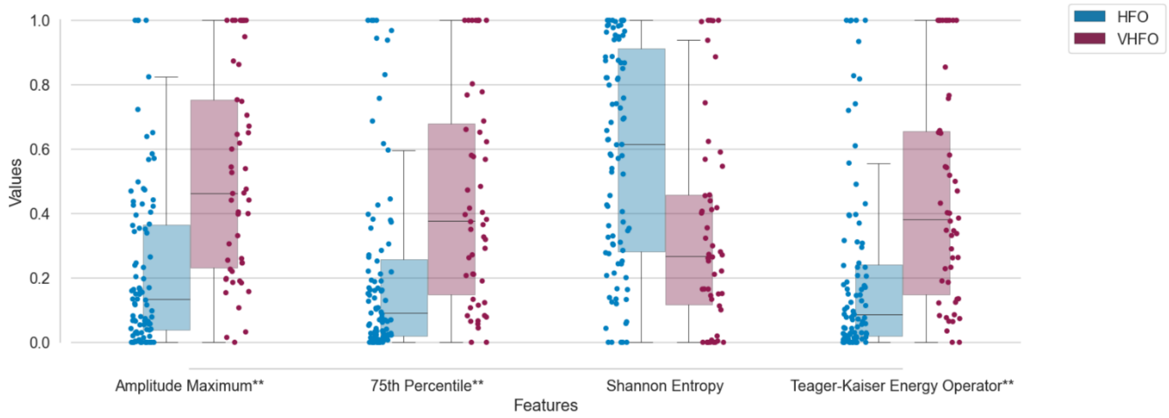

1.0
0.8
0.6
0.4
0.2
0.0
Values
HFO
VHFO
Amplitude Maximum**
75th Percentile**
Shannon Entropy
Teager-Kaiser Energy Operator**
Features

d

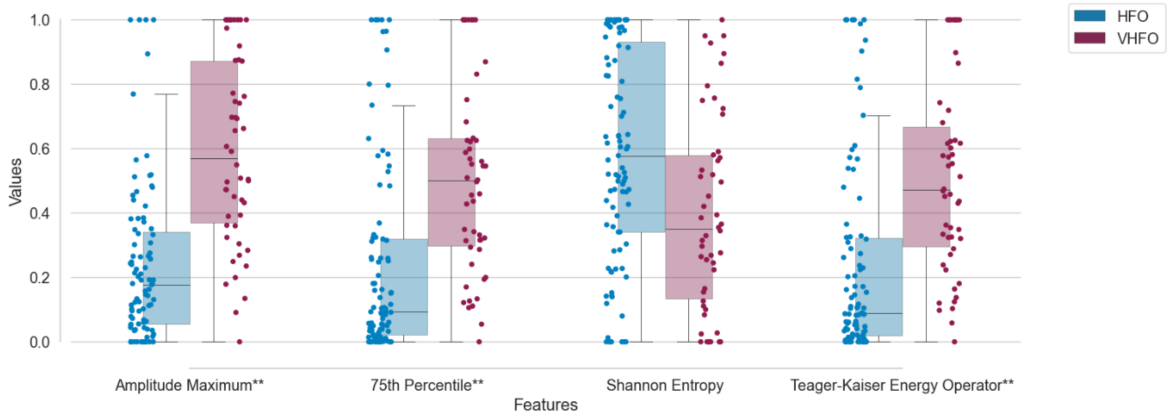

1.0
0.8
0.6
0.4
0.2
0.0
Values
HFO
VHFO
Amplitude Maximum**
75th Percentile**
Shannon Entropy
Teager-Kaiser Energy Operator**
Features

e

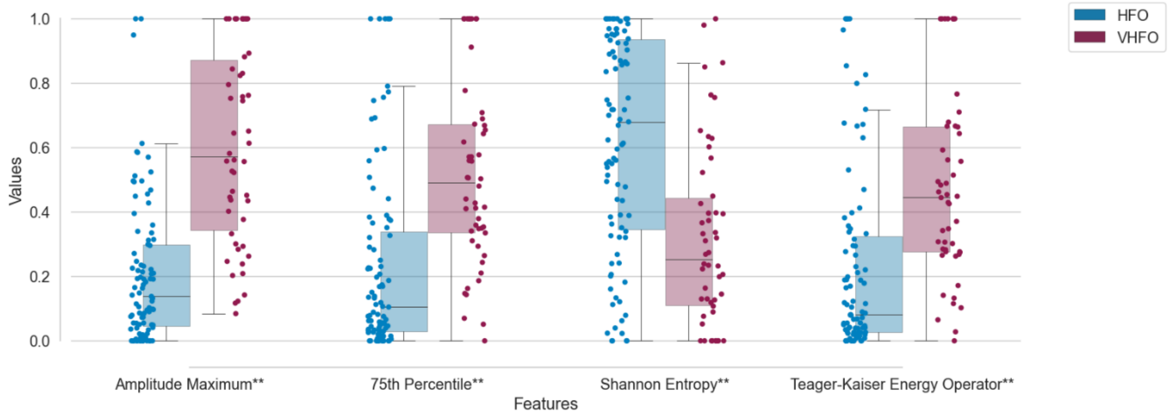

1.0
0.8
0.6
0.4
0.2
0.0
Values
HFO
VHFO
Amplitude Maximum**
75th Percentile**
Shannon Entropy**
Teager-Kaiser Energy Operator**
Features

f

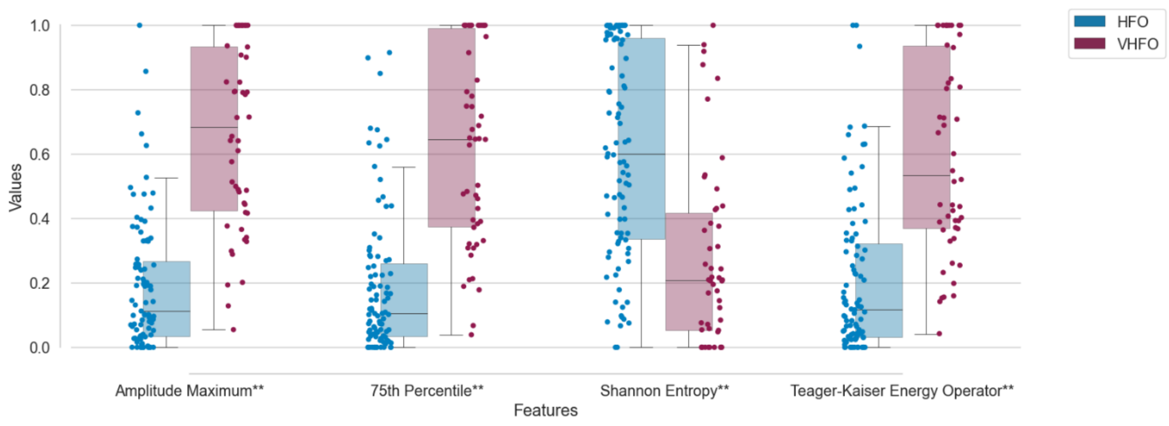

g

Supplementary Figure 1. Boxplots of features of HFO and VHFO channels with 1 kHz sampling frequency in (a) delta, (b) theta, (c) alpha, (d) beta, (e) lower gamma, (f) upper gamma, and (g) ripple frequency band. The boxes show the quartiles of the dataset while the whiskers extend to show the rest of the distribution. Outliers are not visualized. *Results with $0.01<p\leq 0.05$; ** Results with $p \leq 0.01$ and large Cliff's delta are considered significant.

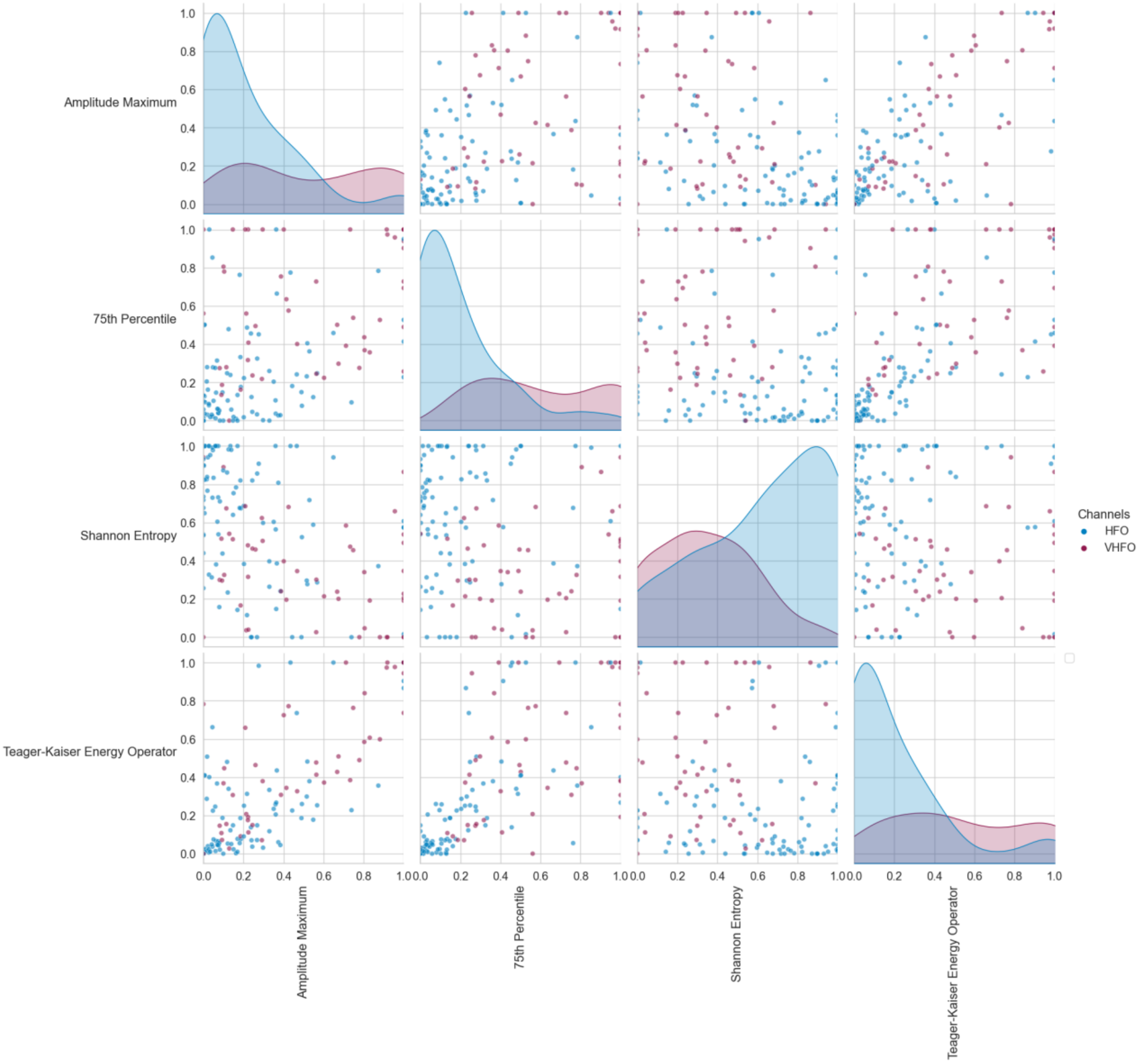

Supplementary Figure 2. Feature values plots displaying the relationships between Amplitude Maximum, 75th Percentile, Shannon Entropy, and Teager-Kaiser Energy Operator calculated in the 0-450 Hz frequency range.
